# What Is Most Important to Family Caregivers When Helping Patients Make Treatment-Related Decisions: Findings from a National Survey

**DOI:** 10.3390/cancers15194792

**Published:** 2023-09-29

**Authors:** Avery C. Bechthold, Andres Azuero, Frank Puga, Deborah B. Ejem, Erin E. Kent, Katherine A. Ornstein, Sigrid L. Ladores, Christina M. Wilson, Christopher E. Knoepke, Ellen Miller-Sonet, J. Nicholas Odom

**Affiliations:** 1 School of Nursing, University of Alabama at Birmingham, Birmingham, AL 35294, USA; abechth@uab.edu (A.C.B.);; 2 Gillings School of Global Public Health, University of North Carolina, Chapel Hill, NC 27599, USA; 3 Center for Equity in Aging, School of Nursing, Johns Hopkins University, Baltimore, MD 21205, USA; 4 Division of Gynecologic Oncology, Heersink School of Medicine, University of Alabama at Birmingham, Birmingham, AL 35233, USA; 5 Division of Cardiology, University of Colorado Anschutz Medical Campus, Aurora, CO 80045, USA; 6 Adult and Child Consortium for Health Outcomes Research and Delivery Science, University of Colorado Anschutz Medical Campus, Aurora, CO 80045, USA; 7 CancerCare, New York, NY 10001, USA; esonet@cancercare.org; 8 Center for Palliative and Supportive Care, University of Alabama at Birmingham, Birmingham, AL 35233, USA

**Keywords:** cancer, caregivers, decision making, oncology, physical well-being, quality of life, treatment preferences, secondary analysis, values, palliative care

## Abstract

**Simple Summary:**

Growing evidence has underscored the importance of assessing patient values, i.e., who or what matters most, in treatment decision making, yet which factors are important to family caregivers remains understudied. Using data from a 2021 national survey of U.S. cancer caregivers, we examined factors that caregivers considered important when partnering with patients during cancer treatment decision making. Caregivers ranked 15 factors on a 4-point Likert-scale from ‘very unimportant’ to ‘very important’. The top 5 most frequently endorsed factors were patient quality of life, physical well-being, length of life, emotional well-being, and the opinions/feelings of oncology team. The endorsement of different factors as ‘very important’, particularly towards ‘opinions/feelings of the oncology team’ varied based on caregiver age, race, gender, and ethnicity. Future work is needed to determine how to best elicit and incorporate caregiver values and preferences into patient treatment decision making.

**Abstract:**

Prioritizing patient values—who/what matters most—is central to palliative care and critical to treatment decision making. Yet which factors are most important to family caregivers in these decisions remains understudied. Using data from a U.S. national survey of cancer caregivers (N = 1661), we examined differences in factors considered very important by caregivers when partnering with patients in cancer treatment decision making by cancer stage and caregiver sociodemographics. Fifteen factors were rated on a 4-point Likert-scale from ‘very unimportant’ to ‘very important.’ Descriptive statistics were used to characterize caregiver factors and tabulate proportions of importance for each. Generalized linear mixed effect modeling was used to examine the importance of factors by cancer stage, and chi-square analyses were performed to determine associations between caregiver sociodemographics and the five most commonly endorsed factors: quality of life (69%), physical well-being (68%), length of life (66%), emotional well-being (63%), and opinions/feelings of oncology team (59%). Significant associations (all *p*’s < 0.05) of small magnitude were found between the most endorsed factors and caregiver age, race, gender, and ethnicity, most especially ‘opinions/feelings of the oncology team’. Future work is needed to determine the best timing and approach for eliciting and effectively incorporating caregiver values and preferences into shared treatment decision making.

## 1. Introduction

As the range of cancer treatments, such as immunotherapy and targeted therapies, continues to evolve and expand and decisions surrounding them become increasingly complex, patient values need to be driving treatment choices [1]. Values are central to patient decision making and adherence [2,3] and are defined as abstract and subjective core beliefs that are context-sensitive and function to guide a person’s preferences, decisions, and actions [4]. Various terms and phrases are often used to refer to values, including factors, priorities, life goals, main goals, ‘what is important’, primary considerations, and ‘what makes life meaningful’ [5]. Simply put, values refer to who or what matters most to a person [6].

To ensure high quality care, clinicians must be adept at applying principles and practices of palliative care, a person- and family-centered approach to care, focused on eliciting and incorporating the values, goals, and preferences of patients and their families into the care plan [7]. This approach is particularly relevant to oncology clinicians who engage with seriously ill individuals from diagnosis through to the end of life. The American Society of Clinical Oncology (ASCO) recommends the concurrent delivery of specialty palliative care alongside oncology care to reduce depression and anxiety, improve symptom management, enhance quality of life for both patients and caregivers, and increase survival [8]. However, the limited availability of palliative care specialists necessitates that all oncology clinicians employ principles of primary (generalist) palliative care. This includes actively and purposefully eliciting patients’ values and operationalizing them into actionable treatment preferences and goals, a key step in shared decision making [8,9,10,11,12].

Although there is a growing body of evidence recognizing that the exploration of patient values and preferences are integral to shared decision making [13], what factors matter most to their unpaid family and friend caregivers in these circumstances remains less clear. Family caregivers, hereafter ‘caregiver’, play a critical decision support role to patients when faced with treatment-related decisions [14,15,16,17] and are often directly impacted by the consequences of these choices, including providing transportation, physical assistance, and emotional support [14]. Cancer caregivers assume various roles in patient’s decision making, such as gathering cancer information, considering hypothetical scenarios related to current and future health states, ensuring a shared understanding among family members and clinicians, facilitating values discussions with the patient, raising collateral decisions stemming from treatment choices (such as the impact on the patient’s ability to work), and making healthcare decisions on behalf of patients who choose to delegate [14].

While the roles that caregivers undertake to support patients in their decision making is becoming clearer, for example, helping to identify the patient’s wishes/needs by considering the side effects of a specific treatment and how they may impact the patient’s quality of life [18], less is known about what caregivers value as important in this role and what factors caregivers prioritize when partnering in cancer treatment-related decision making. To address this gap, we analyzed data from a recent U.S. national survey of caregivers to explore the most highly endorsed factors among all caregivers who reported having ever been involved in making a treatment-related decision regarding their care recipient with cancer. For clarity, the term ‘factors’ will be used hereafter, as opposed to ‘values’, since this term was used in the survey. We hypothesized that the most highly endorsed factors would differ between caregivers of patients with early- versus late-stage cancers. Further, we explored associations between caregiver sociodemographic characteristics and the extent to which factors were endorsed as very important.

## 2. Methods

Following Kelley and colleagues’ survey conduct and reporting guidelines (Supplement S1) [19], this cross-sectional survey study used data from a U.S. online survey of family caregivers of patients with cancer (N = 2703) conducted and distributed for online completion by Cancer*Care*^®^ between February and July 2021. Cancer*Care* is a national 401(c)(3) nonprofit organization founded in 1944 that provides free, professional support services to individuals with cancer and their families. Caregivers were eligible for inclusion, if they (1) provided unpaid care/support to a family member or friend with cancer and who does not have to live with them, (2) had been providing care/support to this person for at least 6 months, and (3) were aged >18 years. Of the 2703 caregivers who completed the survey (Supplement S2), our analysis included 1661 caregivers who reported having been involved in making treatment-related decisions regarding their care recipient with cancer. These treatment-related decisions included involvement in deciding on the treatment plan, where to receive treatment, when to receive treatment, whether to stop cancer treatment completely, and obtaining a second opinion on the treatment plan.

Details of the survey design have been previously described [20,21]. In brief, the 20–30 min survey was developed in partnership with Cancer*Care*’s caregiving service staff and national research experts in family caregiving (including JNO and EM-S) to describe the role of cancer caregivers in patient decision making and to assess their support needs. Survey items were reviewed by a Cancer*Care* advisory board (five professional patient advocates) and piloted by a Cancer*Care* social worker and other staff who provide counseling to cancer caregivers. The original Cancer*Care* survey contracted with a market panel research company (PureSpectrum, Westlake Village, CA, USA) to identify respondents from consumer research panels. PureSpectrum used an advanced respondent scoring system (PureScore 1.0) to authenticate participants across online market research panels and block any duplicate or fraudulent activity. The use of market research panels to invite survey respondents prohibited the calculation of a survey response rate. Caregivers were defined in the survey as individuals who self-identified as a family or friend caregiver of an individual with cancer and reported involvement in making decisions. These individuals actively provided unpaid support and did not have to reside in the same home. PureSpectrum collected, sorted, and validated all survey responses. To aid in the geographical representativeness of the survey, the sample had approximately 25% participants in the Northeast, Midwest, Southeast, and Southwest/West, respectively. Participants were not paid for participation. The study was deemed exempt by the University of Alabama at Birmingham Institutional Review Board as all data included were de-identified.

## 3. Measures

### 3.1. Demographic and Clinical Characteristics

Caregiver respondents self-reported sociodemographic (e.g., age, gender, race, ethnicity, education, geographic location, caregiver–patient relationship) and clinical information (e.g., cancer type and stage, length of time providing care). Cancer stage was reported by first asking what type of cancer the patient was diagnosed with (‘What type of cancer was the person diagnosed with?’) and the cancer stage, including remission, at the time of taking the survey (‘What stage is the person’s cancer as of today?’). If caregivers selected leukemia, they were diverted to a leukemia-specific staging question.

### 3.2. Items to Measure Treatment Decision Making and Caregiver Factors

Caregiver involvement in cancer treatment decision making was ascertained using an item set that asked respondents to select all decision areas they had ever been involved in since providing support to their care recipient with cancer. Five of those items asked specifically about decisions related to treatment: ‘Deciding where to get treatment’, ‘Deciding whether to begin treatment’, ‘Deciding on the treatment plan (e.g., surgery, radiation, chemotherapy, immunotherapy, targeted therapy)’, ‘Getting a second opinion on the treatment plan’, and ‘Deciding whether or not to stop cancer treatment completely’.

**Figure 1:**
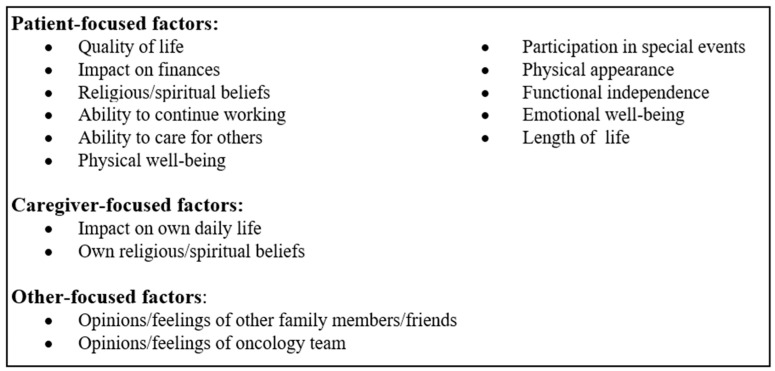
Factors endorsed by family caregivers as impacting treatment-related decision making.

The next section of the survey consisted of a ‘Decision Deep Dive’ section where respondents were asked to answer questions about their role and challenges faced within each decision area they remembered most clearly. Within the Deep Dive section, the importance of caregiver factors in treatment-related decision making was assessed with the item: ‘When you were participating in making this [treatment] decision, how important to you were these factors regarding the person with cancer?’. A total of 15 factors were ranked on a 4-point Likert scale ranging from ‘very unimportant’ to ‘very important’. See Figure 1.

## 4. Statistical Methods

Descriptive statistics were used to characterize caregiver’s demographic and clinical characteristics and tabulate proportions of reported importance for each of the factors in treatment-related decision making. An overall test of difference in proportions of importance across stages was conducted using a generalized linear mixed effect model with random effects for subject to account for clustering of responses within individual, and as fixed effects, indicator variables for factors, cancer stage, and an interaction between these two. Multiple degree-of-freedom interaction test was used to assess whether the importance of factors depends on cancer stage. Following the interaction test, post hoc Pearson’s chi-square tests of association between the proportions of importance for each factor and cancer stage were conducted at a target 10% false discovery rate level. To help assess the magnitude of differences, Cramer’s V was computed and interpreted using Cohen’s guidelines (small~0.1, medium~0.3, large~0.5) [22] and the proportion of importance for each factor by cancer stage was compared to the overall proportion (all stages combined). Pearson’s chi-squared tests were performed to determine associations between caregiver sociodemographic characteristics and the top five factors caregivers endorsed as ‘very important’ during cancer treatment decision making. Cramer’s V was computed to aid in interpretation and a multiple testing correction was conducted at a target 10% false discovery rate level. All analyses were conducted using IBM SPSS Statistics 29, besides the multiple test corrections, which were conducted using R version 4.2.0.

## 5. Results

Sociodemographic and clinical characteristics of caregivers are shown in Table 1. Caregivers (N = 1661) represented a range of ages with individuals aged 35–54 comprising the largest bracket (51%). Just over half were female (51%) and most were White (80%). Approximately 16% of the sample was Hispanic/Latino. The educational backgrounds of caregivers varied, with approximately 64% having a 4-year college degree or additional educational attainment, and a majority reporting a total household income of USD 75,000 or more (66%). Most caregivers lived in urban settings (84%). Caregivers reported being a family member (63%), friend (24%), or spouse/partner (12%) to the care recipient and the majority had provided care for between 1 and 3 years (76%). Patients had a wide range of solid tumor (83%) and hematologic cancers (14%) with breast (22%), lung (15%), and prostate (13%) cancers representing the most common types. Most patients either had early-stage (44%) or late-stage cancer (49%), although a small proportion was in remission (5%). Most patients lived separately from their caregivers in a home/apartment (53%), assisted living facility (4%), or nursing home (6%).

**Figure 2:**
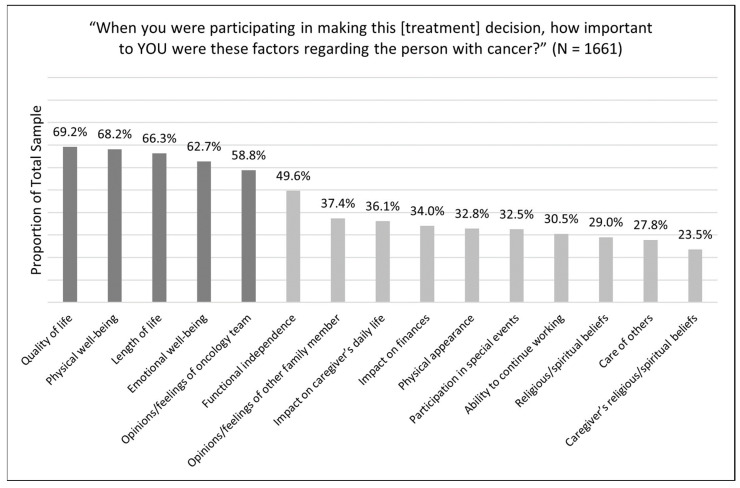
Factors endorsed as very important by caregivers during treatment decision making. Dark bars illustrate caregiver factors endorsed by greater than 50% of the total sample.

Figure 2 depicts the overall ranking of factors endorsed by caregivers as ‘very important’ during cancer treatment-related decisions.

Of the 15 factors, patient quality of life was the one most often endorsed as ‘very important’ (69%, n = 1150). Other factors endorsed as very important by over half of the caregiver sample included patient physical well-being (68%, n = 1133), patient length of life (66%, n = 1101), patient emotional well-being (63%, n = 1041), and the opinions/feelings of the oncology team (59%, n = 977). Although the ranking of these top five factors changed slightly when proportions of ‘important’ and ‘very important’ were aggregated together, the top five factors remained the same. Similar to the top-ranked factors, the ranking of the bottom-ranked factors changed slightly when proportions of ‘important’ and ‘very important’ were considered together; however, the bottom five factors remained the same. All factors, besides the top six factors, were endorsed in a small proportion of 37% or less of the sample.

Table 2 presents a comparison of caregiver endorsement of each factor as ‘very important’ by cancer stage, including ‘in remission’. The interaction test of stage group by factor (F(28, 22,652) = 1.6, *p* = 0.023) indicated that the pattern of endorsement across stage groups varied across factors. Compared to all stages combined, caregivers of early-stage cancer patients endorsed 14-out-of-15 factors as ‘very important’ at a higher proportion. The only value endorsed at a lower proportion was patient physical appearance. In contrast, caregivers of patients with late-stage cancer endorsed 13-out-of-15 values as ‘very important’ at a lower proportion compared to all stages combined. Caregivers of patients in remission endorsed all 15 factors as ‘very important’ at a lower proportion compared to all stages combined. After conducting a multiple testing correction, *p*-values <0.02 were considered statistically significant at a 7.5% false discovery rate level. Significant associations of small magnitude were observed between patient cancer stage and patient physical well-being (*p* = 0.012; Cramer’s V = 0.074), patient length of life (*p* = 0.016; Cramer’s V = 0.071), opinions/feelings of the oncology team (*p* = 0.007; Cramer’s V = 0.078), patient ability to continue working (*p* = 0.005; Cramer’s V = 0.081), and patient ability to care for others (*p* = 0.020; Cramer’s V = 0.070).

Results from the chi-square tests exploring associations between caregiver sociodemographic characteristics and the extent to which factors were endorsed as very important are depicted in Table 3. After multiple test correction, *p*-values < 0.024 were considered statistically significant at an 11% false discovery rate level. Significant associations of small magnitude were observed between caregiver age and patient quality of life (*p* < 0.001; Cramer’s V = 0.122), patient physical well-being (*p* < 0.001; Cramer’s V = 0.130), patient emotional well-being (*p* < 0.001; Cramer’s V = 0.091), and the opinions/feelings of the oncology team (*p* < 0.001; Cramer’s V = 0.137); caregiver gender and the opinions/feelings of the oncology team (*p* = 0.006; Cramer’s V = 0.079); caregiver race and the opinions/feelings of the oncology team (*p* = 0.009; Cramer’s V = 0.085); and caregiver ethnicity and patient physical well-being (*p* = 0.004; Cramer’s V = 0.071), patient length of life (*p <* 0.001; Cramer’s V = 0.101), and the opinions/feelings of the oncology team (*p* < 0.001; Cramer’s V = 0.088).

## 6. Discussion

Using data from 1661 family caregivers of individuals with cancer, we explored the most highly endorsed factors among all caregivers who reported having ever been involved in making a treatment-related decision regarding their care recipient with cancer. Our analysis revealed that the following factors were most important to caregivers: quality of life (69.2%), physical well-being (68.2%), length of life (66.3%), emotional well-being (62.7%), and opinions/feelings of the oncology team (58.8%). Significant associations of small magnitude were found between these highest-rated factors and caregiver age, race, gender, and ethnicity, most especially the opinions/feelings of the oncology team. Given the significant decision support that caregivers provide to their care recipients in cancer treatment decision making [14,15,16,17,23,24], this study provides important data on what factors may drive their roles and engagement. These findings can assist clinicians in conducting focused assessments that explore and integrate these caregiver factors during shared decision making.

The caregivers of patients with early-stage cancers more often endorsed physical well-being, length of life, and the opinions/feelings of the oncology team as very important factors in treatment decision making, when compared to caregivers of patients with late-stage cancers. However, the relative importance of these factors remained the same across stages (i.e., quality of life as the top value followed by physical well-being, length of life, etc.). One possible explanation for the increased importance of these factors in the context of early cancer is that caregivers may initially perceive the cancer as curable and heavily rely on the opinions of the oncology team due to limited understanding of the disease, treatments, and care management [25]. Over time, as the cancer progresses or reoccurs, caregivers may perceive it as incurable and rely more heavily on their own developed understanding of the disease and its management [25], leading to a diminished emphasis on these factors. This possible explanation is consistent with the existing literature [26,27,28], which finds that patients with advanced cancer and their caregivers express a preference for patient-led decision making. Our findings suggest that although physical well-being, length of life, and the opinions/feelings of the oncology team may be perceived as less important in late-stage cancers, they still rank among the most important factors considered during treatment decision making. Therefore, it is important for clinicians to elicit and consider these factors during shared decision making regardless of cancer stage.

Older caregivers (>55 years) more often endorsed quality of life, physical well-being, and emotional well-being as very important factors in treatment decision making. This preference aligns with previous studies indicating that older age is associated with an increased tendency to decline potentially life-prolonging measures in the context of advanced cancer, including anticoagulants, antibiotics, artificial hydration, blood transfusions, chemotherapy, cardiopulmonary resuscitation/mechanical ventilation, or feeding tubes [29,30]. Moreover, a Canadian study of 380 patients diagnosed with advanced cancer and referred to a regional palliative medicine consultative program found that older individuals expressed a preference for conservative palliative management (i.e., focused on maximizing comfort, dignity, and quality of life), whereas younger individuals tended to favor aggressive medical management (i.e., focused on sustaining or prolonging life) [28]. Palliative care, which aims to relieve symptoms and psychological distress and may be delivered alongside curative treatments [7], is particularly relevant for adolescents and young adults with cancer and their caregivers [31]. However, integration remains suboptimal among young adults (1/5 or <20%), especially among younger (<25 years), male, and non-Hispanic White patients, as indicated by a U.S. national inpatient sample from 2016–2019 [31]. These findings highlight a gap in the provision of palliative care and emphasize the need for additional support in these populations.

Older caregivers more often endorsed the opinions/feelings of the oncology team as very important factors in treatment decision making compared to younger caregivers. Older age is associated with a higher level of trust in medical professionals, which influences an individual’s willingness to seek/accept care and their openness in sharing sensitive information [32,33]. In contrast, younger adults with cancer and their caregivers tend to place more trust in information from family, friends, or the internet, rather than medical professionals [34,35]. Recognizing that younger individuals are more likely to turn to online sources, clinicians should be prepared to share reputable sources of information to supplement caregivers’ information needs. Common information needs reported by caregivers include information on treatment (35.6%), disease (35.6%), and side effects (26.5%) [36].

White caregivers more often endorsed the opinions/feelings of the oncology team as very important factors in treatment decision making when comparing White and Black caregivers. The existing scientific literature [35,37] has extensively documented the presence of mistrust in healthcare clinicians and the healthcare system among non-Hispanic Black and Hispanic/Latino individuals. Medical mistrust is detrimental to relationships between the healthcare team and patient/family [37]. For example, individuals who report medical mistrust are less likely to communicate pertinent medical information; engage in beneficial health behaviors, such as medication adherence and screening; and are more likely to experience a worse symptom burden, poorer quality of life, and lower satisfaction with care [37,38]. Addressing all forms of discrimination in healthcare settings, including racial/ethnic, language, and income/insurance, is crucial to reducing disparities in health outcomes [37]. At the individual level, clinicians can obtain additional training in areas such as antiracism, implicit bias, linguistic, cultural competency, and structural competency, and foster partnership by engaging patients and caregivers in shared decision making [37]. Additionally, racial and ethnic matching between clinicians and patients/families has been found to promote positive health behaviors and treatment outcomes [37].

Non-Hispanic White caregivers more often endorsed physical well-being, length of life, and the opinions/feelings of the oncology team as very important factors in treatment decision making, when comparing Non-Hispanic White and Hispanic/Latino caregivers. These variations in the importance assigned to different factors could be attributed to the influence of Hispanic cultural values. One such value is fatalism, which entails the belief that events are beyond an individual’s control and outcomes are unchangeable. Previous research has demonstrated the role of fatalism in cancer treatment among Hispanics and Latinos [39]. Specifically, fatalism has been associated with lower adherence to cancer screening and the belief that death is inevitable following a cancer diagnosis [40,41,42]. Consequently, it is plausible that Hispanic/Latino caregivers are less likely to prioritize length of life as a significant factor when making treatment decisions. Additional cultural values, such as familism, can drive the importance of factors in treatment decision making. For example, a recent qualitative study found that Latino cancer patients preferred the involvement of family members in advance care planning [43]. Thus, Hispanic/Latino caregivers may be more likely to prioritize family input over the opinions/feelings of the oncology team when making decisions.

Understanding and acknowledging these cultural values is imperative for healthcare providers to engage with Hispanic/Latino caregivers more effectively and promote consideration of important decision making factors when discussing treatment options. Suggested steps to address culturally competent support for cancer caregivers include cultural responsiveness training, standardized language training for clinicians, and incentive pay for fluency [44]. The use of professional interpretation services versus ad hoc interpretation have also been found to improve trust and satisfaction among Latino patients with limited English proficiency [44]. Further research should explore the interplay between cultural values, decision making, and caregiver experiences to develop culturally responsive interventions that meet the diverse needs of cancer caregivers from various backgrounds. Specifically, studies that go beyond the traditional caregiver–care–recipient dyad to examine caregiving networks and family decision making are needed. There is also a need for studies focused on additional cultural factors—such as minority stress, acculturation, and language—that may influence decision making among diverse caregiving populations.

### 6.1. Limitations

There were several limitations in this study. First, the study data were cross-sectional, and hence we are unable to examine potential changes in factors over time. Longitudinal work could address this by documenting important caregiver factors across the disease trajectory from diagnosis through bereavement, including cancer recurrence. Second, our results are limited to the factors reported by caregivers. While our findings are novel in that they report on caregiver factors rather than those of patients, future studies should consider reporting on both patient and caregiver factors to examine concordance. It remains uncertain how treatment decision making proceeds when patient and caregiver factors are discordant and how such situations impact both patient and caregiver outcomes. Third, our sample predominantly consisted of non-Hispanic White (80%), highly educated (college graduate = 34%; postgraduate = 37%), high-income (33%), and urban-dwelling individuals (84%). Compared to national caregiving demographics from the Caregiving in the U.S. 2020 report [45], our sample had a slightly higher proportion of non-Hispanic White individuals (61%) and college (21%) and graduate degree (14%) attainment, lower household income (47% ≥USD 75,000), and a similar proportion of urban-dwelling individuals (88%). Thus, this limits the generalizability of our findings. Future work is needed to explore the factors of diverse groups since values—who/what matters most—develop through a combination of genetic factors, such as temperament and socialization by various social groups (e.g., family, peers) and social institutions (e.g., schools, religious communities) [46]. Similarly, response rates were unable to be computed due to the use of market research panels to acquire the sample. This adds additional caution to the generalizability of these findings. Cancer*Care* decided not to use survey responses from caregivers in its own client database to not bias the results by enlisting individuals who had previously used their services since they would have already been engaged in thinking about the process of caregiving. Finally, caregivers were not provided with specific definitions for each factor (e.g., quality of life), which may have led to different interpretations when rating each factor. A lack of standardized definitions may partially explain why ‘quality of life’ was the most frequently endorsed factor, as it can have different meanings to different people without further specification.

### 6.2. Clinical Implications

Our findings have several implications for the clinical setting in the context of clinical decision making. Clinicians need to recognize that family caregivers assume a wide range of roles in a patient’s decision making and are often proximally impacted by the implications of a treatment choice. What matters most to patients and caregivers should be elicited and revisited across the disease trajectory, particularly during transition periods such as diagnosis, beginning and concluding treatments, recurrence, and progression to advanced disease. In clinical practice, a closed-ended or domain-driven approach, whereby individuals select/rate factors from a predefined list/categories, may be more practical for eliciting these factors compared to an open-ended approach, whereby individuals freely describe/list factors [47]. For example, a values elicitation/clarification tool like the ‘What Matters Most—Structured Tool (Revised)’ [48] can be adapted to identify the factors that impact cancer decision making. This tool was originally developed for older adults with multimorbidity and involves selecting up to three values within each category: ‘functioning’, ‘enjoying life’, and ‘connecting’. Cancer patients and caregivers may be asked to separately select or rate personally relevant factors from a short list either before or during their clinic visit. By incorporating principles and practices of primary palliative care into routine clinical practice, oncology clinicians can provide holistic care that aligns with values, goals, and preferences [49]. Further research is needed to determine the optimal timing (prior to or during clinic visits) and method (alone or facilitated by another individual) for eliciting patient and caregiver factors.

## 7. Conclusions

Our results from a large national survey found that quality of life, physical well-being, length of life, emotional well-being, and opinions/feelings of oncology team were the factors most often considered by caregivers during cancer treatment decision making. Moreover, we found that caregiver characteristics such as age, race, gender, and ethnicity were associated with the endorsement of different values as very important, particularly towards the ‘opinions/feelings of the oncology team.’ These findings offer valuable insight into what factors might be driving caregiver roles and engagement and can assist clinicians in effectively applying principles and practices of primary palliative care—that is, eliciting and incorporating caregiver values and preferences into the decision making process. Future work is needed to determine the optimal timing and method of eliciting what matters most to caregivers and how to best integrate their values and preferences into shared treatment decision making.

## Figures and Tables

**Table 1:** Demographic characteristics of caregivers reporting involvement in treatment-related decisions for a patient with cancer.

Characteristic	Participated in Cancer Treatment Decision Making and Responded to ‘Deep Dive’ Questions, N = 1661
	n (%)
Caregiver age	
18–34	476 (28.7)
35–54	839 (50.5)
55 and older	343 (20.7)
Missing ^1^	3 (0.2)
Caregiver gender	
Male	793 (47.7)
Female	851 (51.2)
Trans woman/man or gender non-conforming	16 (1.0)
Missing ^1^	1 (0.1)
Caregiver race	
White	1322 (79.6)
African American/Black	183 (11.0)
Asian	95 (5.7)
Alaskan Native, American Indian, Native Hawaiian, or Pacific Islander	18 (1.1)
Missing ^1^	43 (2.6)
Hispanic/Latino	
Yes	262 (15.8)
No	1395 (84.0)
Missing ^1^	4 (0.2)
Caregiver education	
Postgraduate degree	498 (30.0)
Some postgraduate	109 (6.6)
College graduate (4 y)	565 (34.0)
Vocational/Technical School (2 y)	79 (4.8)
Some college	250 (15.1)
High school graduate or less	158 (9.5)
Missing ^1^	2 (0.1)
Caregiver employment status	
Working full time (>30 h per week)	1153 (69.4)
Working part time (<30 h per week)	217 (13.1)
Other (student, retired)	171 (10.3)
Not employed	89 (6.8)
Missing ^1^	7 (0.4)
Caregiver total household income	
≥USD 75,000	1093 (65.8)
<USD 75,000	548 (33.0)
Missing ^1^	20 (1.2)
Location	
Urban	1394 (83.9)
Rural or small town	213 (12.8)
Missing ^1^	54 (3.3)
Caregiver-patient relationship	
Family member (sibling, child, parent, aunt/uncle, grandparent, cousin)	1043 (62.8)
Friend	405 (24.4)
Spouse/partner	200 (12.0)
Missing ^1^	13 (0.8)
Length of time providing care	
Up to 1 year	530 (31.9)
1 to <3 years	736 (44.3)
3 to <5 years	204 (12.3)
5 or more years	191 (11.5)
Patient’s cancer type	
Solid tumor cancers ^2^	1382 (83.2)
Breast	363 (26.3)
Lung	254 (18.4)
Prostate	218 (15.8)
Colon/rectal	111 (8.0)
Other	436 (31.5)
Hematologic cancers ^3^	234 (14.1)
Missing ^1^	45 (2.7)
Patient’s cancer stage (solid tumor cancers)	
In remission	48 (2.9)
1–2	629 (37.9)
3–4	720 (43.4)
Patient’s cancer stage (hematologic cancers)	
In remission	26 (1.6)
1–2	107 (6.4)
3–4	91 (5.5)
Missing ^1^ (solid and hematologic)	40 (2.4)
Patient’s insurance type	
Employer insurance	791 (47.6)
Medicare	436 (26.2)
Medicaid	156 (9.4)
Marketplace exchange	123 (7.4)
No insurance	49 (3.0)
Missing ^1^	106 (6.4)
Patient’s living situation	
Living with caregiver	597 (35.9)
Living apart from caregiver	1046 (63.0)
Missing ^1^	18 (1.1)

^1^ Missing indicates ‘missing’, ‘do not know’, ‘prefer not to say’, ‘prefer not to answer’, or ‘none of the above’. ^2^ Solid tumor cancer types: bladder, brain, breast, colon/rectal, gynecologic, head and neck, kidney, lung, melanoma, pancreatic, prostrate, thyroid. ^3^ Hematologic cancer types: leukemia, lymphoma, multiple myeloma.

**Table 2:** Caregiver factors ranked by importance when making cancer treatment-related decisions by cancer stage.

Factors ^1^	All Stages, N = 1661	Early Stage, n = 736	% ^2^	Late Stage, n = 811	% ^2^	In Remission, n = 74	% ^2^	*p*-Value ^3^
	%	n (%)		n (%)		n (%)		
Quality of life	69.2	526 (71.5)	+2.3	552 (68.1)	−1.1	45 (60.8)	−8.4	0.095
2 Physical well-being	68.2	524 (71.2)	+3.0	546 (67.3)	−0.9	41 (55.4)	−12.8	0.012 *
3 Length of life	66.3	518 (70.4)	+4.1	522 (64.4)	−1.9	44 (59.5)	−6.8	0.016 *
4 Emotional well-being	62.7	477 (64.8)	+2.1	496 (61.2)	−1.5	46 (62.2)	−0.5	0.330
5 Opinions/feelings of oncology team	58.8	468 (63.6)	+4.8	453 (55.9)	−2.9	42 (56.8)	−2.0	0.007 *
6 Functional independence	49.6	382 (51.9)	+2.3	388 (47.8)	−1.8	34 (45.9)	−3.7	0.228
7 Opinions/feelings of other family members/friends	37.4	297 (40.4)	+3.0	293 (36.1)	−1.3	21 (28.4)	−9.0	0.055
8 Impact on caregiver’s daily life	36.1	269 (36.5)	+0.2	269 (33.2)	+1.0	17 (23.0)	−6.4	0.448
9 Impact on finances	34.0	240 (32.6)	+2.5	280 (34.5)	−0.8	16 (21.6)	−11.0	0.042
10 Physical appearance	32.8	267 (36.3)	−0.2	301 (37.1)	+1.7	22 (29.7)	−11.2	0.073
11 Participation in special events	32.5	253 (34.4)	+1.9	261 (32.2)	−0.3	16 (21.6)	−10.9	0.076
12 Ability to continue working	30.5	219 (29.8)	+4.1	234 (28.9)	−2.6	21 (28.4)	−7.5	0.005 *
13 Religious/spiritual beliefs	29.0	255 (34.6)	+0.8	226 (27.9)	−0.1	17 (23.0)	−0.6	0.914
14 Ability to care for others	27.8	225 (30.6)	+2.8	219 (27.0)	−0.8	12 (16.2)	−11.6	0.020 *
15 Caregiver’s religious/spiritual beliefs	23.5	178 (24.2)	+0.7	189 (23.3)	−0.2	17 (23.0)	−0.5	0.911

*p*-values ≤ 0.020 are considered statistically significant at a 7.5% false discovery rate level. Interaction test of stage group by factor: F(28, 22,652) = 1.6, *p* = 0.023. Percentages might not add to 100% due to missing data regarding stage for 26 respondents (1.5%). ^1^ Factors are patient-focused unless indicated otherwise. ^2^ Positive indicates a factor was reported as ‘very important’ at a higher proportion compared to all stages. Negative indicates a factor was reported as ‘very important’ at a lower proportion compared to all stages. ^3^ Pearson’s chi-square was used to determine *p*-values and Cramer’s V. * Small magnitude of difference using Cohen’s guidelines (small~0.1, medium~0.3, large~0.5).

**Table 3:** Associations between the top five factors reported as very important and caregiver sociodemographic factors.

Characteristic	Quality of Life		Physical Well-Being		Length of Life		Emotional Well-Being		Opinions/Feelings of Oncology Team	
	n (%)	*p*-Value ^1^	n (%)	*p*-Value ^1^	n (%)	*p*-Value ^1^	n (%)	*p*-Value ^1^	n (%)	*p*-Value ^1^
Caregiver age										
18–34 (n = 476)	296 (62.2)	<0.001 *	289 (60.7)	<0.001 *	295 (62.0)	0.027	276 (58.0)	<0.001 *	236 (49.6)	<0.001 *
35–54 (n = 839)	583 (69.5)		573 (68.3)		580 (69.1)		520 (62.0)		502 (59.8)	
55 and older (n = 343)	269 (78.4)		268 (78.1)		223 (65.0)		242 (70.6)		236 (68.8)	
Caregiver gender										
Male (n = 793)	530 (66.8)	0.055	526 (66.3)	0.134	509 (64.2)	0.069	474 (59.8)	0.051	453 (57.1)	0.006 *
Female (n = 851)	610 (71.7)		598 (70.3)		583 (68.5)		557 (65.5)		520 (61.1)	
Other, trans woman/man or gender non-conforming (n = 16)	9 (56.3)		9 (56.3)		8 (50.0)		9 (56.3)		4 (25.0)	
Caregiver race										
White (n = 1322)	923 (69.8)	0.040	915 (69.2)	0.052	884 (66.9)	0.109	827 (62.6)	0.503	802 (60.7)	0.009 *
African American/Black (n = 183)	130 (71.0)		122 (66.7)		125 (68.3)		118 (64.5)		102 (55.7)	
Asian (n = 95)	56 (58.9)		58 (61.1)		57 (60.0)		55 (57.9)		44 (46.3)	
Other, Alaskan Native, American Indian, Native Hawaiian, or Pacific Islander (n = 18)	9 (50.0)		8 (44.4)		8 (44.4)		9 (50.0)		7 (38.9)	
Caregiver ethnicity										
Hispanic/Latino (n = 262)	166 (63.4)	0.024	159 (60.7)	0.004 *	145 (55.3)	<0.001 *	150 (57.3)	0.042	128 (48.9)	<0.001 *
Non-Hispanic/Latino (n = 1395)	982 (70.4)		973 (69.7)		954 (68.4)		891 (63.9)		848 (60.8)	
Employment										
Full-time (n = 1153)	789 (68.4)	0.140	784 (68.0)	0.613	775 (67.2)	0.135	721 (62.5)	0.074	676 (58.6)	0.838
Part-time (n = 217)	147 (67.7)		144 (66.4)		145 (66.8)		136 (62.7)		124 (57.1)	
Unemployed (n = 113)	89 (78.8)		83 (73.5)		77 (68.1)		82 (72.6)		70 (61.9)	
Other, retired, student, or prefer not to say (n = 178)	125 (70.2)		122 (68.5)		104 (58.4)		102 (57.3)		107 (60.1)	
Caregiver total household income										
≥USD 75,000 (n = 1093)	770 (70.4)	0.254	746 (68.3)	0.823	738 (67.5)	0.237	677 (61.9)	0.180	649 (59.4)	0.651
<USD 75,000 (n = 548)	371 (67.7)		377 (68.8)		354 (64.6)		358 (65.3)		319 (58.2)	
Caregiver-patient relationship (The patient is the caregiver’s…)										
Spouse/partner (n = 200)	139 (69.5)	0.845	137 (68.5)	0.763	136 (68.0)	0.423	127 (63.5)	0.980	121 (60.5)	0.687
Family (n = 1043)	727 (69.7)		716 (68.6)		699 (67.0)		655 (62.8)		605 (58.0)	
Friend (n = 405)	276 (68.1)		270 (66.7)		258 (63.7)		254 (62.7)		243 (60.0)	
Location										
Rural or small town (n = 213)	157 (73.7)	0.171	151 (70.9)	0.387	137 (64.3)	0.452	128 (60.1)	0.319	130 (61.0)	0.516
Urban (n = 1394)	963 (69.1)		947 (67.9)		933 (66.9)		887 (63.6)		818 (58.7)	

*p*-values < 0.024 are considered statistically significant at an 11% false discovery rate level. Percentages might not add to 100% due to missing data on each characteristic (location: n = 54, 3.3%; race: n = 43, 2.6%; income: n = 20, 1.2%; all other characteristics: <0.8%). ^1^ Pearson’s chi-square was used to determine *p*-values and Cramer’s V. Factors are listed by the factor most reported as very important from left to right. * Small magnitude of difference using Cohen’s guidelines (small ~0.1, medium ~0.3, large ~0.5).

## Data Availability

The data that support the findings of this study are available from Cancer*Care* upon reasonable request.

## Supplementary Materials

The following supporting information can be downloaded at: https://www.mdpi.com/article/10.3390/cancers15194792/s1, Supplement S1: Good practice in the conduct and reporting of survey research reporting guidelines; Supplement S2: Cancer*Care*’s Caregiver Decision-Making survey.
