# Supplementary material for: What Is Most Important to Family Caregivers When Helping Patients Make Treatment-Related Decisions: Findings from a National Survey
Source: Cancers (Basel). 2023 Sep 29;15(19):4792. doi: 10.3390/cancers15194792 (PMC10572058; doi:10.3390/cancers15194792)
Supplement: Supplementary file 1 [file cancers-15-04792-s001.zip › Supplement S2.pdf]

SECTION 1: SCREENING

Let's begin by making sure our survey is right for you.

1. First, please select the state you live in from the dropdown menu below.

   (STATE DROPDOWN)

2. In the past 12 months, have you provided unpaid care/support to someone close to you who has cancer? This person can be a family member or friend and does not have to live with you?

   Yes 1
   No.................. 2 **TERMINATE**

Since their cancer diagnosis, how long have you been providing care/support to this person? This does not have to be a continuous period of time.

Less than 6 months **TERMINATE**
Between 6 months to less than 1 year ....
1 to less than 3 years 3
3 to less than 5 years 4
5 to less than 7 years 5
7 to less than 9 years 6
9 to less than 10 years 6
10 years or longer.. 7

SECTION 1: SCREENING

Next, please complete the following demographic questions for both you and the person for whom you provide unpaid care and support. We only ask these questions for classification purposes. They allow us to look at the results by different kinds of caregivers and people with cancer, to see how they may be similar. Again, all of this information is kept strictly confidential.

3. Please indicate your gender and the person with cancer's gender below.

	Your Gender	Person with cancer's Gender
Male	❑	❑
Female	❑	❑
Gender diverse (non-conforming, transgender)		❑
Prefer not to say	❑	❑

4. What is your age and what is the age of the person you care for with cancer?

	Your Age	Person with cancer's Age	
Under 18	❑	❑	**TERMINATE**
18 to 24	❑	❑	
25 to 34	❑	❑	
35 to 44	❑	❑	
45 to 54	❑	❑	
55 to 64	❑	❑	
65 to 74	❑	❑	
75 or older	❑	❑	
Prefer not to say	❑	❑	

SECTION 1: SCREENING

5. Are either you or the person with cancer of **Hispanic, Latino or Spanish origin**?

	You	Person with cancer
Yes	❑	❑
No	❑	❑
Prefer not to say	❑	❑

6. Please indicate your **race/ethnicity** below. Again, please answer for both you and the person with cancer.

	Your Race/Ethnicity	Person with cancer's Race/Ethnicity
White/Caucasian	❑	❑
Black/African American	❑	❑
Asian or Pacific Islander	❑	❑
Alaskan Native/American Indian	❑	❑
Other	❑	❑
Prefer not to say	❑	❑

7. Have you or the person with cancer been diagnosed with the COVID-19 virus?

	You	Person with cancer
Yes	❑	❑
No	❑	❑
Don't Know	❑	❑

8. In which of the following areas, if any, have you **ever** been involved in making decisions regarding the person with cancer you care for? Please read the list carefully and select all that apply.

DECISIONS RELATED TO DIAGNOSIS

Seeking a second opinion on the diagnosis .... ❑

Testing the tumor for genetic mutations (bio-marker testing)

DECISIONS RELATED TO TREATMENT

Deciding where to get treatment .... ❑

Deciding when to begin treatment .... ❑

Deciding on the treatment plan (e.g., surgery, radiation, chemo, immunotherapy, targeted therapy)... ❑

Getting a second opinion on the treatment plan .... ❑

Deciding whether or not to be in a clinical trial... ❑

Determining what medications to take to treat the symptoms and side effects of treatment? .... ❑

Deciding whether or not to stop cancer treatment completely .... ❑

Deciding whether or not to get alternative therapy instead of standard treatment.. ❑

DECISIONS RELATED TO DOCTOR/TREATMENT LOCATION

Switching to another doctor or cancer center .... ❑

DECISIONS RELATED TO EMERGENCY CARE

Deciding if the person with cancer should go to the emergency room due to cancer symptoms or side effects .... ❑

FINANCIAL DECISIONS

Deciding how to pay for treatment .... ❑

Deciding whether or not to get paid home health care .... ❑

OTHER DECISIONS:

Deciding whether or not to get palliative or comfort care .... ❑

Deciding whether or not to get rehabilitation.. ❑

Deciding whether or not to get hospice or end of life care .... ❑

I have not been involved in making any of these types of decisions .... ❑ END SURVEY

Thank you. We are very interested in your responses to this survey. Please click on the forward button to continue.

SECTION 2: PERSON WITH CANCER PROFILE

In this next section, we would like to know more about **the person with cancer** you provide care/support for.

9. Where does the person with cancer live?

   Home/Apartment with me.. ❑
   Home /Apartment, not with me................................ ❑
   Nursing Home .... ❑
   Assisted living facility .... ❑
   None of the above.. ❑

   ASK IF 'NOT HOME/APARTMENT WITH ME'

10. How far away from you does the person live from you? Please estimate the time it takes you to travel there by car.

    Less than 5 minutes .... ❑
    5 to 15 minutes... ❑
    16 to 30 minutes... ❑
    31 to 45 minutes... ❑
    46 to 60 minutes... ❑
    Longer than an hour but less than 2 hours .... ❑
    Longer than 2 hours .... ❑
    Don't know .... ❑

11. What is your relationship to the person with cancer?

    The person with cancer is….
    My spouse/partner ....❑
    My mother/father ....❑
    My child ....❑
    My brother/sister
    My grandparent ....❑
    Someone in my extended family (aunt/uncle/cousin/etc.)................................❑
    A friend ....❑
    Other ....❑

12. Are you the health care proxy for this person? That is, you make health care decisions for the person with cancer if they are not able to speak for themselves.

    Yes
    No
    Not sure

SECTION 2: PERSON WITH CANCER PROFILE

13. What type of cancer was the person diagnosed with?

Bladder..............................❑
Brain..................................❑
Breast................................❑
Cervical ....❑
Colon or Rectal ....❑
Endometrial/Uterine
Head and Neck ....❑
Kidney ....❑
Chronic Lymphocytic Leukemia (CLL)
Leukemia (other types) ......❑
Lung ....❑
Lymphoma ....❑
Melanoma ....❑
Multiple Myeloma ....❑
Ovarian..............................❑
Pancreatic ....❑
Prostate.............................❑
Stomach ....
Thyroid ....❑
Endometrial/Uterine ....❑
Other (Specify)..................❑

14. How long ago was the person **first diagnosed** with cancer? Your best estimate is fine.

Within the last six months .... ❑
7 to 12 months ago .... ❑
13 to 18 months ago .... ❑
19 to 24 months ago .... ❑
More than 2 years ago .... ❑

IF LEUKEMIA IN Q.13, SKIP TO Q.16
15. What stage is the person's cancer as of today?

Stage 1 a small tumor that has **not** spread to other parts of the body ....❑
Stage 2: a larger tumor that has grown deeply into the tissues ....
Stage 3: it **has spread** beyond the tumor but not to nearby organs ....❑
Stage 4: it **has spread** (metastasized) to distant parts of the body or organs .....❑
In remission: the signs and symptoms have disappeared but not necessarily the entire disease..❑
Not sure/don't know ....❑

ASK IF LEUKEMIA

16. What stage is the person's cancer as of today?

- Stage 0, 1 or 2: high lymphocyte counts, swollen lymph nodes, and/or spleen and/or liver ❑
- Stage 3 or 4: high levels of white blood cells, likely anemia ❑
- In remission: the signs and symptoms have disappeared but not necessarily the entire disease ❑
- Not sure/don't know ❑

17. How would you describe the person with cancer's current medical treatment? Select all that apply.

- Watchful waiting ❑
- They are in active treatment ❑
- They completed active treatment and are on maintenance therapy ❑
- They are no longer in treatment ❑
- They are in a clinical trial
- Other (Please specify) ❑
- I am not sure ❑

18. Which of the following types of treatment, if any, has the person with cancer experienced? Please check all that apply.

- Surgery ❑
- Chemotherapy ❑
- Radiation ❑
- Oral Drug therapy ❑
- Immune or biologic therapy (designed to strengthen the body's natural defense system ❑
- Alternative, non-traditional treatment ❑
- Complementary treatment (such as acupuncture, massage therapy) ❑
- Other (Please specify) ❑
- I am not sure ❑
- None ❑

SECTION 3: CAREGIVER EXPERIENCE

In this next section, we would like to know more about **your experiences caring/supporting the** person with cancer.

19. How many other people in addition to yourself have provided unpaid care/support for this person with cancer?

No one else provides unpaid care besides me 1
1 other person (in addition to me) 2
2 other people (in addition to me) 3
3 or more other people (in addition to me) 4

20. On average, how often do you provide care/ support to them? This could be emotional support or any other activity where you are helping the person with cancer cope with their illness. Your best estimate is fine.

Daily.. 1
Several Times Per Week 2
Once Per Week 3
2 to 3 Times Per Month 4
Monthly 5
Less Often Than Monthly 6

21. On a **typical day,** how many hours do you spend providing care/ support for the person with cancer? Your best estimate is fine.

Less than 1 hour.. 1
1 to 2 hours.. 2
3 to 4 hours.. 3
5 to 6 hours.. 4
7 to 10 hours.. 5
More than 10 hours 6

22. In your opinion, how often does this person with cancer need help to communicate effectively with their cancer doctors or nurses?

All of the time... 1
Most of the time 2
Sometimes... 3
Not usually... 4
Never 5

23. In your opinion, how often does the person with cancer dress, bathe, and prepare and eat food without the help of a caregiver?

All of the time 1

Most of the time 2

Sometimes 3

Not usually 4

Never 5

SECTION 4: DECISION DEEP DIVE

In this next section, we would like to ask you some more questions about some of the decision areas you noted you were involved in.

PROGRAMMING: SHOW ONLY DECISIONS respondent is involved in FROM Q.8

24. Of the following decisions you indicated you were involved in, which ones do you **remember most clearly**? Please select up to three.

25. You indicated you were involved in a decision related to (PIPE IN DECISION AREA). In your own words, please describe the situation and your involvement in as much detail as you can. Please try to be as specific as possible.

---

26. Has the person with cancer given you official permission to speak directly with their healthcare team? (A HIPAA release)

27. Please describe the part you played in this particular decision.

The person with cancer made the decision. I was an observer and played a supportive role. ❑
I made the decision. The person with cancer and other family and/or friends provided their input. ❑
The person with cancer and I made the decision together. We both agreed on the best choice. ❑
The healthcare team made the decision. The person with cancer and I provided our input but left the final decision up to the healthcare team. ....❑
Other; Please describe: ....❑

28. Who else was involved in making this decision? Check all that apply.

RANDOMIZE

Family/close friends..❑
Faith or spiritual advisors..❑
The doctor and/or other clinical team members ....❑
Other (specify)...❑

29. Not including members of the clinical team, how many people were part of this decision making group?

1 to 3...❑
4 to 6 ....❑
7-10 ....❑
More than 10 ....❑

30. When this decision was being made, where did **you** look for helpful information? (check all that apply)

RANDOMIZE ALL EXCEPT THE LAST BULLET

The person with cancer's clinical care team .... ❑

Friends or family .... ❑

Medical professionals (other than the person with cancer's care team) ❑

The internet .... ❑

Educational materials given to us by the person with cancer's clinical care team ❑

Social Media .... ❑

Government agencies or organizations .... ❑

Non-profit organizations for caregivers or people with cancer.. ❑

Clergy or spiritual advisor .... ❑

Never looked for help or information .... ❑

31. Thinking about this decision, how much do you agree or disagree with the following statements?

Strongly agree	Somewhat agree	Somewhat disagree	Strongly disagree

The information you found or were given was hard to understand
The right decision was made
I regret the choice that was made
I would go for the same choice if I had to do it over again
The choice was harmful to the person with cancer

32. In your experience as a caregiver of a person with cancer, has a nurse, doctor or social worker asked you about what you needed to help you share in making decisions regarding this person with cancer?

Yes .... ❑
No .... ❑
Not sure .... ❑

33. In your own words, please tell us what was challenging about making this decision. Please be as specific as possible when answering.

34. And how challenging did each of the following make it for **you** to arrive at a decision in this situation? Please answer using the scale shown.

Not At All Challenging To Make Decision	Not Very Challenging To Make Decision	Somewhat Challenging To Make Decision	Very Challenging To Make Decision	Extremely Challenging To Make Decision	Not Applicable

- Not everyone on the care team agreed
- Some team members didn't agree with the doctor's recommendation
- I didn't have enough info to make this decision
- I didn't understand how the treatment would work
- I didn't understand the out of pocket costs of treatments

- I didn't know caregiver responsibilities for each of the treatment options
- I didn't know how treatment(s) would affect the person with cancer's physical condition
- I didn't know how treatments would affect the person with cancer's quality of life
- I didn't understand the treatment schedules
- I didn't understand the risks and benefits of treatment
- I didn't know the wishes of the person with cancer

35. When you were participating in making this decision, how important to YOU were these factors regarding the person with cancer?

Very Unimportant	Unimportant	Important	Very Important	Not Applicable

RANDOMIZE

- The person's quality of life
- The impact of the choice on the person's finances
- The person's religious/spiritual beliefs
- The person being able to continue working
- The person's ability to take care of others (e.g., children)
- The person's physical well-being
- The impact of the decision on being able to participate in special events (e.g., weddings, births, travel plans)
- Changes in the person's appearance such as hair loss or weight gain/loss
- The person being able to function independently in activities of daily living (e.g., feeding, bathing, getting dressed)
- The impact of the decision on the person's emotional well-being
- The person's length of life
- The opinions and feelings of other family members and friends
- The opinions of the of the person's cancer doctor and healthcare team
- The consequences of this decision on **my** own day to day life
- **My** own religious and/or spiritual beliefs

36. Have you and the person with cancer discussed any particular wishes they have about the care they would want to receive at the end of life?

Yes ....❑
No ....❑
Not sure ....❑

SECTION 5: CAREGIVER ATTITUDES

37. How much does each statement describe how you feel about providing care for your friend or family member with cancer? (randomize)

Not at all **Somewhat** **Quite a bit** **Completely**

- I feel completely overwhelmed
- I feel useful and needed
- Caring for this person with cancer gives my life a purpose and a sense of meaning.
- I would not have chosen the situation I'm in, but I get satisfaction out of providing care
- Family and friends don't give me enough help
- I believe caring for this person is a religious calling
- I feel trapped by this person's illness
- In general, I feel competent as a caregiver
- Since becoming a caregiver I have become more aware of my inner strengths
- Since becoming a caregiver I have become more self-confident
- Since becoming a caregiver I have grown as a person
- Since becoming a caregiver I have learned to do things I didn't do before
- I trust myself to make the best decisions in caring for my close friend/family member
- I am very satisfied with the care the doctors/clinical team provide
- I believe the person I care for values my input about treatment
- I believe the doctors/clinical team value my input about treatment
- I need a break from being a caregiver
- I feel like there is no end in sight to my caregiving
- I am not able to care for myself or my family because I am spending so much time as a caregiver
- No one is taking care of me

38. When you are or were providing care for your close friend or family member with cancer, how often do/did you feel? (randomize)

Never **Rarely** **Sometimes** **Quite Frequently** **Nearly Always**

- Informed enough about the person with cancer's illness.
- Bothered that other family members have not shown interest in taking care of the person with cancer
- Encouraged that communication in my family has improved
- Satisfied with the support you get from your family or friends
- Sad for what my life together with this person would have been like without cancer
- Scared that it's not safe to leave the person alone
- Stressed between the pressures of caregiving and meeting other family or work responsibilities
- Sad or angry that you have lost control of your life
- Tense that you have a loss of privacy and/or personal time
- Stressed that family members don't agree about the best treatment for the person with cancer
- Sad for what my life would be like without this person's cancer

SECTION 6: IMPACT OF FACTORS ON DECISIONS

39. Have you felt that the support you and the person with cancer have received for making cancer related decisions by your doctor or healthcare team has been negatively affected by any of the following [check all that apply]:

    Age,
    Race,
    Language,
    Education level,
    Health or disability,
    Internet access,
    Political affiliation,
    Body weight,
    Insurance type or lack of insurance,
    Income level,
    Religion,
    Sexual orientation,
    Gender/sex,
    Other (please explain) ________________________

SECTION 7: SERVICES

40. How unhelpful or helpful would the following services be to you when you are involved in helping make treatment decisions for a close friend or family member with cancer

RANDOMIZE

	Not helpful	Helpful	Very helpful
On-line support group of caregivers in similar situations as me			
On-line support group of people with the same health conditions as my friend/family member			
Free consultation with an oncology nurse			
Educational materials about cancer from a trusted source			
Free consultation with an oncology social worker			
Free consultation with an oncology doctor			
Videos about treatment decision making			
Worksheets that help guide decision making			
Role playing how to share in treatment decision making			
Information on sharing decisions about cancer treatment			

SECTION 8: ANXIETY GAD-2 and DEPRESSION PHQ-2

41. Over the **last 2 weeks**, how often have you been bothered by the following problems?

	Not At All	Several Days	More than half the days	Nearly Every Day
Feeling nervous, anxious, or on edge				
Not being able to stop or control worrying				
Little interest or pleasure in doing things				
Feeling down, depressed or hopeless				

SECTION 9: DEMOGRAPHICS

You are almost finished! Just a few more demographic questions about you and the person with cancer.

Again, we ask these only for classification purposes.

ASK ONLY IF THE RESPONDENT HAS NOT ANSWERED SPOUSE/PARTNER IN Q. 12

42. Please indicate your **marital status** and the marital status of the person with cancer below.

	Your Marital Status	Person with cancer's Marital Status
Married	❑	❑
Domestic partnership	❑	❑
Single, living with a partner	❑	❑
Single (including divorced, widowed or separated)	❑	❑
Prefer not to say	❑	❑

43. Please indicate your **employment status** and the employment status of the person with cancer below. Check all that apply

	Your Employment Status	Person with cancer's Employment Status
Working full time (30+ hours per week)	❑	❑
Working part time (less than 30 hours per week)	❑	❑
Retired	❑	❑
Student (part time or full time)	❑	❑
Not employed, looking for work	❑	❑
Not employed, not looking for work	❑	❑
Stopped working due to cancer		
Prefer not to say	❑	❑

44. Please indicate the **highest level of education** you and the person with cancer have completed below.

	Your Education	Person with cancer's Education
Some High School or Less	❑	❑
High School Graduate	❑	❑
Vocational/Technical School (2 Year)	❑	❑
Some College	❑	❑
College Graduate (4 Year)	❑	❑

Some Post-Graduate	❑	❑
Post Graduate Degree	❑	❑
Prefer not to say	❑	❑

45. And what type of **medical insurance** do you have, if any.

	Your Medical Insurance	Person with cancer's Medical Insurance
Medicare	❑	❑
Medicaid	❑	❑
Employer insurance	❑	❑
Marketplace exchange (Affordable Care Act)	❑	❑
Veteran's insurance		
Other	❑	❑
No Insurance	❑	❑

46. Please indicate which of the following categories best describes **your total annual household income** before taxes.

	Your Household Income
Under $15,000	❑
$15,000 to $29,999	❑
$30,000 to 49,999	❑
$50,000 to 74,999	❑
$75,000 to 99,999	❑
$100,000 to 149,999	❑
$150 000 and greater	❑
Prefer not to answer	❑

ASK IF PERSON WITH CANCER AND CAREGIVER LIVE IN SEPARATE HOUSEHOLDS (from Q.10)

47. Please indicate which of the following categories best describes your (PIPE IN) **total annual household income** before taxes. Your best estimate is fine.

	Person with cancer's Household Income
Under $15,000	❑
$15,000 to $29,999	❑

$30,000 to 49,999 ❑
$50,000 to 74,999 ❑
$75,000 to 99,999 ❑
$100,000 to 149,999 ❑
$150 000 and greater ❑
Prefer not to answer ❑

48. Have you or the person with cancer been diagnosed with COVID-19? Please select all that apply.

I have ❒
The person with cancer has ❒
No, neither of has been diagnosed ❒

49. What else would you like to share about your experiences in helping your close friend or relative with cancer to make health and treatment decisions?

____
____

50. Would you like to receive a copy of the report from this study when it is published?

Yes ❒
No ❒

If yes, please provide your name and email address……….

____

51. Finally, would you be willing to participate in a follow up survey and/or discussion about your caregiving experience?

Yes ❒
No ❒

If yes, please provide your name and email address……….

____
